# The Role of Nrf2 in Skeletal Muscle on Exercise Capacity

**DOI:** 10.3390/antiox10111712

**Published:** 2021-10-27

**Authors:** Yu Kitaoka

**Affiliations:** Department of Human Sciences, Kanagawa University, 3-27-1 Rokkakubashi, Kanagawa-ku, Yokohama 221-8686, Kanagawa, Japan; kitaoka@kanagawa-u.ac.jp; Tel.: +81-45-481-5661

**Keywords:** Nrf2, oxidative stress, skeletal muscle, mitochondria

## Abstract

Nuclear factor erythroid 2-related factor 2 Nfe2l2 (Nrf2) is believed to play a crucial role in protecting cells against oxidative stress. In addition to its primary function of maintaining redox homeostasis, there is emerging evidence that Nrf2 is also involved in energy metabolism. In this review, we briefly discuss the role of Nrf2 in skeletal muscle metabolism from the perspective of exercise physiology. This article is part of a special issue “Mitochondrial Function, Reactive Oxygen/Nitrogen Species and Skeletal Muscle” edited by Håkan Westerblad and Takashi Yamada.

## 1. Introduction

Reactive oxygen species (ROS) are produced in skeletal muscles both at rest and during physical activity, mainly by mitochondria. Under physiological conditions, these ROS are buffered by the cellular antioxidant system to prevent the accumulation of oxidative damage. However, when the redox balance is disrupted, oxidative stress is known to be associated with metabolic disorders and skeletal muscle dysfunction. Indeed, elevated levels of oxidative stress markers can be observed in skeletal muscles in neuromuscular diseases, such as muscular dystrophy (Duchenne [1], limb-girdle [2], and facioscapulohumeral [3]), and metabolic myopathies (McArdle disease [4], Pompe disease [5], and mitochondrial disease [6]). Furthermore, physical inactivity, obesity, and aging-related muscle dysfunctions are linked to the increase in oxidative stress [7,8,9]. In contrast, there is growing evidence that ROS production promotes beneficial adaptations in skeletal muscles, including mitochondrial biogenesis. From this point of view, several studies have demonstrated that antioxidant supplementation hampers exercise training induced muscle adaptations [10,11]. These hormesis (or mitohormesis) effects of exercise have drawn a great deal of attention from researchers in both health and sports sciences [12,13]. As ROS production is called a “double-edged sword”, it is important to consider its pros as well as cons.

## 2. Nrf2 as a Master Regulator of the Response to Oxidative Stress

Nuclear factor erythroid 2-related factor 2 Nfe2l2 (known as Nrf2) is a transcription factor that is believed to be the key regulator of the antioxidant response [14,15]. Under quiescent/homeostatic conditions, Nrf2 is constantly degraded via the Kelch-like ECH-associated protein 1 (Keap1) mediated ubiquitin-proteasome pathway. Under stress conditions, however, Nrf2 translocates into the nucleus for binding to the antioxidant response element (ARE) of the target cytoprotective genes. Studies using Nrf2-deficient mice on a C57BL/6 background show that Nrf2 is important for antioxidant enzymes in skeletal muscles (Table 1). Among the established downstream targets of Nrf2 identified in other tissues, one of the most significantly down-regulated proteins in Nrf2 knockout (KO) mice is NAD(P)H quinone oxidoreductase 1 (NQO1), which is involved in the detoxification process [16].

However, Nrf2 deficiency has minimal effect on the expression of its downstream antioxidant enzymes, except NQO1, in the skeletal muscles of young mice [17,18]. This may be due to the fact that Nrf2 plays only a limited role in skeletal muscle redox status under basal/unstressed conditions. Nrf2 has been shown to be essential for antioxidant and cytoprotective gene induction in response to muscle contraction [19], as well as inactivity caused by surgical denervation [20]. In aged mice, the effects of Nrf2 deficiency on antioxidant proteins seem to be much greater, indicating a lack of compensatory adaptation to age-associated ROS accumulation [18,21,22,23]. Indeed, loss of Nrf2 induces the oxidative stress marker 4-HNE in whole muscle homogenates [18,21] and mitochondrial fractions [23] of aged skeletal muscles. Similar observations have been reported in cardiac muscle [24]. 

## 3. Nrf2 and Mitochondrial Function

Mitochondria are energy-producing organelles, and hence their content in skeletal muscles is strongly correlated with endurance exercise capacity [25,26]. As byproducts of oxidative metabolism, mitochondrial ROS are produced in complexes I and III, and this ROS production might lead to cumulative mitochondrial/cellular damage. Given that mitochondria have their own DNA, which lacks protective histones, their quality control mechanism is essential for the maintenance of mitochondrial function [27]. Mitochondria are highly dynamic organelles that constantly undergo fusion and fission. The fusion process allows the sharing of nucleic acids as well as metabolic substrates, while the fission process enables damaged mitochondria to be detached and degraded [28].

It has been demonstrated that acute exposure to ROS induces fragmentation of the mitochondrial network, with reduced number of fusion and fission events in C2C12 myoblasts [29,30]. Therefore, it seems to be logical to assume that ablation of Nrf2 affects skeletal muscle mitochondria and, particularly, exacerbates aging-associated mitochondrial dysfunction. Since previous studies have shown that Nrf2 deficiency leads to impaired mitochondrial respiration in mouse embryonic fibroblasts, as well as various tissues including liver and brain [31,32,33], this review focused on research on skeletal muscles. Table 2 illustrates the effects of Nrf2 deficiency on mitochondria in mouse skeletal muscles. Although the loss of Nrf2 does not have much impact on skeletal muscle mitochondrial volume and morphology in young mice, impaired mitochondrial dynamics and increased percentage of abnormal mitochondria are observed in an age-dependent manner [34]. Increased ROS production in aged Nrf2 KO mice is observed in both permeabilized muscle fibers [22] and isolated mitochondria [23]. Interestingly, even in young mice, the lack of Nrf2 reduces state 4 respiration and increased ROS emission in intermyofibrillar mitochondria, but these changes are not observed in subsarcolemmal mitochondria [17], indicating that the effect of Nrf2 deficiency depends on subcellular localization of mitochondria. Recently, mass spectrometry analysis revealed that Nrf2 KO changes the expression of over a hundred proteins, including several proteins essential for normal mitochondrial function, in skeletal muscle specific transgenic mouse model [35]. The decline in complex I expression in Nrf2 KO muscle is consistent with the findings of another study, that examined respiratory function and showed reduced complex I-linked oxygen consumption rate [22]. Further studies are needed to probe the differences in respiratory function depending on the substrate.

Although the number of studies is limited, it is worth noting that Nrf2 has been reported to mediate exercise-induced mitochondrial biogenesis in skeletal muscles. Lack of Nrf2 impairs treadmill running induced increases in citrate synthase (CS) activity and mitochondrial DNA copy number [36] and attenuates voluntary wheel running induced increase in cytochrome-c oxidase (COX) activity [17]; these are well known markers of mitochondrial content. Another study reported that Nrf2 deficiency abolishes the effects of hypoxia preconditioning for 48 h on exercise-induced CS activity and oxidative phosphorylation (OXPHOS) protein levels [37]. Despite this, not all mitochondrial proteins are affected by the loss of Nrf2, and intriguingly, mitochondrial transcription factor A (TFAM) expression has been shown to be higher in Nrf2 KO mice [16]. These studies suggest that Nrf2 is required for complete exercise-induced mitochondrial adaptation in skeletal muscles, but the molecular mechanisms of how Nrf2 interacts with other signaling pathways already known to be involved in mitochondrial biogenesis remain to be elucidated.

## 4. Nrf2 Signaling in Response to Physical Activity

Regular physical activity has numerous health benefits, while a sedentary lifestyle leads to reduced tolerance to stressors and increased risk of chronic diseases [38]. It is widely known that exercise acutely produces ROS in skeletal muscles as energy demand rapidly increases [12,39]. Hence, the question is whether acute exercise/chronic training changes Nrf2 expression. Previous studies involving exercise intervention using mouse models are listed in Table 3. Several studies have demonstrated that acute treadmill running increases Nrf2 transcription [36,40] and ARE binding activity with translocation into the nucleus [17,41,42] in mouse skeletal muscles.

An acute bout of treadmill exercise for 60–150 min transiently increases Nrf2 mRNA levels in mouse skeletal muscles, followed by a return to the baseline levels by 18 h [36,40]. At the protein level, incremental exercise tests to exhaustion [17,42] and for 6 h, but not 1 h of running at 20 m/min [41], activates Nrf2 signaling, suggesting that longer exercise duration induces higher responses. In C2C12 cells using electrical pulse stimulation model, the response of Nrf2 has been shown to depend on the intensity and time of muscle contraction [19]. More recently, muscle stimulation of both high (100 Hz) and low (50 Hz) frequency has been found to activate Nrf2-ARE binding in stimulated muscles. Surprisingly, Nrf2-ARE activation was also observed in contralateral unstimulated muscles after high intensity stimulation, suggesting that Nrf2 signaling is activated in peripheral tissues in response to exercise [43].

Despite the fact that exercise training increases antioxidant enzymes [44], there are conflicting reports regarding the effects of chronic training on Nrf2 protein content in whole muscle homogenates in mice [17,42,45]. This may be due to the difference in training modes (voluntary or forced treadmill), period (4 wk or 6 wk), recovery before muscle sampling (24 h or 48 h), and strain of mice (C57BL/6 or ICR/CD-1). Importantly, Merry et al. reported that commercially available Nrf2 antibodies were unable to detect the single band corresponding to Nrf2 in muscle lysates at correct molecular weight, that was not seen in lysates from Nrf2 KO mice [36]. Also, it should be noted that there has been a misconception about the molecular weight of Nrf2 [46]. Given the short half-life of Nrf2, which is considered to be less than 20 min [47], downstream targets of Nrf2, rather than Nrf2 itself, should be examined as chronic adaptations. 

In a human study, muscle Nrf2 protein levels were reported to be higher in active old subjects than in subjects with a sedentary lifestyle [9]. In agreement with mouse studies, an acute bout of exercise, examining both endurance (30 min of continuous cycling) and Tabata (4 min of supramaximal interval exercise) protocols, up-regulated Nrf2 mRNA expression in human skeletal muscles [48]. Similarly, both the two 30 min cycling trials (high-intensity interval and constant workload) elicited an increase in Nrf2 levels in nuclear fractions of the peripheral blood mononuclear cells from young subjects [49]; however, the increase in nuclear Nrf2 induced by 30 min cycling at 70% VO_2_max was not observed in older subjects [50]. Nrf2 signaling is activated by acute high intensity interval exercise in humans, but the exposure to a hypoxic environment does not seem to augment the response [51,52]. Future studies are needed to clarify the exercise conditions (mode, intensity, and duration) required for increasing Nrf2 expression in human skeletal muscles.

## 5. Nrf2 and Muscle Contractile Function

Skeletal muscle characteristics of Nrf2 KO mice are summarized in Table 4. In accordance with mitochondrial adaptations, Nrf2 deficiency does not dramatically influence skeletal muscle mass [17,20] or cross-sectional area [34,53] in young mice, whereas aged Nrf2 KO mice show a trend of reduction in these indices compared with age-matched wild-type (WT) mice [18,22,23,34]. Likewise, Nrf2 has negligible impact on denervation-induced [20] and streptozotocin-induced [54] muscle atrophy. When mice have access to a running wheel in their cage, there is no difference in daily running distance between Nrf2 KO mice and WT mice. Nrf2 deficiency decreases maximal grip strength and inverted grid hanging duration in aged mice, but not in young mice [22,34,42]. These studies suggest that that Nrf2 deficiency affects muscle mass and physical function mainly in an age-dependent manner.

However, in isolated muscle, it is important to note that Nrf2 KO mice show a significantly reduced force generation with time, and a greater rate of fatigue, irrespective of age [17,22,35]. Exercise capacity, as evaluated by an incremental treadmill test to exhaustion, has also been found to be impaired in Nrf2 KO mice [34,35,53], though some studies failed to find any significant changes compared with WT mice [17,42]. These conflicting results are not only because of animal age, but also due to differences in experimental protocols, such as treadmill incline, speed patterns, criteria for exhaustion, and acclimatization to the treadmill. Furthermore, it should be noted that while most of the studies referred to in this review used global Nrf2 KO mice, some used skeletal muscle specific [42], and also inducible [35], KO models. Another study demonstrated that improvement in aerobic capacity after forced treadmill running, which is observed in WT mice, is not observed in Nrf2 KO mice [36]. Recently, Keap1 deficiency, which activates the Nrf2 pathway, has been shown to enhance exercise capacity as well as force generation in mouse skeletal muscles [35,55]. Moreover, Sulforaphane treatment, which is known to activate Nrf2, increases running distance in an exhaustive treadmill running test in WT mice [53], and alleviates the reduction in grip strength and exercise capacity in mdx mice, the most widely used animal model of Duchenne muscular dystrophy [56]. Similarly, treatment with phytochemicals known to activate Nrf2 (marketed as Protandim) has been found to enhance exercise-training induced skeletal muscle adaptations. Interestingly, it does not blunt the positive effects of exercise, which are observed in response to supplementation with exogenous antioxidants such as vitamin C [57]. Taken together, these results indicate that Nrf2 is important for skeletal muscle function and exercise performance, possibly beyond the role of the master regulator of antioxidant genes. 

## 6. Summary and Perspectives

Given the rapid changes in energy demand during exercise, it seems obvious that antioxidant systems regulated by Nrf2 are important for athletic performance. In skeletal muscle, Nrf2 signaling is activated by acute exercise, and this response is required for exercise-mediated enhancement of antioxidant proteins and, at least in part, mitochondrial biogenesis. It has been reported that the loss of Nrf2 reduces skeletal muscle contractile function, while activation of Nrf2 enhances exercise performance in mice. However, Nrf2 deficiency does not necessarily change the expression of muscular antioxidant enzymes under basal conditions especially in young mice, indicating the existence of compensatory mechanisms. Considering that there are various types and sources of ROS produced in skeletal muscle during exercise, it will be necessary to clarify how each of them is involved in athletic performance and adaptation of energy metabolism to exercise training. In particular, it should be emphasized that subcellular localization of ROS production and scavenging needed to be taken into account, as glycogen and mitochondria are distinctly distributed within skeletal muscle cells [58,59]. Furthermore, in addition to the cellular antioxidant systems, effects of dietary antioxidants should also be considered. Lastly, it is important to highlight that data obtained from mice cannot be directly applied to humans, especially highly trained athletes.

## Figures and Tables

**Table 1:** Effects of Nrf2 deficiency on skeletal muscle antioxidants in C57/BL6 mice.

Reference	Animal Age	Protein Abundance/Enzyme Activity
Miller et al., [18]	2 months	↑ GCS; ↓ NQO1, G6PD; ↔ CAT, GSR, SOD1, SOD2
	24 months	↑ SOD2; ↓ NQO1, CAT, GCS, GSR, G6PD; ↔ SOD1
Narasimhan et al., [21]	>23 months	↓ NQO1, CAT, GPX1, G6PD, SOD1; ↔ GSR, SOD2
Crilly et al., [17]	3 months	↓ NQO1; ↔ HO-1, GPX1, G6PD
Ahn et al., [22]	24 months	↓ GPX4, GSR, GSTA3, GSTM1, GSTP1, PRDX1, TXN1, TXNRD1; ↔ GPX1, SOD1, SOD2
Kitaoka et al., [23]	22 months	↓ CAT; ↔ Total SOD

↑: Increased, ↓: Decreased, ↔: Unchanged vs. age-matched wild-type mice. Abbreviations: NQO1, NAD(P)H quinone oxidoreductase 1; CAT, catalase; HO-1, heme oxygenase 1; GCS, glutamyl cysteine synthase; GPX, glutathione peroxidase; GSR, glutathione reductase; GSTA3, glutathione S-transferase A3; GSTP1, glutathione S-transferase pi 1; G6PD, glucose 6-phosphate dehydrogenase; PRDX1, peroxiredoxin 1; SOD: superoxide dismutase, TXN1, thioredoxin 1; TXNRD1, thioredoxin reductase 1.

**Table 2:** Effects of Nrf2 deficiency on skeletal muscle mitochondria in C57/BL6 mice.

Reference	Summary of Effects
Crilly et al., [17]	Decreased state 4 respiration and increased ROS emission in isolated IMF mitochondria
Merry et al., [36]	Abolished exercise training-induced increase in mtDNA copy number and CS activity
Ahn et al., [22]	Reduced complex I-linked respiration and increased ROS production in PMF
Kitaoka et al., [23]	Increased ROS production and oxidative stress markers in isolated mitochondria
Huang et al., [34]	Decreased mtDNA copy number and impaired mitochondrial morphology
Wang et al., [37]	Abolished effects of hypoxia preconditioning on CS activity and OXPHOS protein levels
Gao et al., [35]	Downregulated proteins involved in mitochondrial health/remodeling

Abbreviations: ROS, reactive oxygen species; IMF, intermyofibrillar; mtDNA, mitochondrial DNA; CS, citrate synthase; PMF, permeabilized muscle fibers; OXPHOS, oxidative phosphorylation.

**Table 3:** Effects of exercise on Nrf2 expression in mouse skeletal muscles.

Reference	Study Design	Nrf2			
		mRNA	Protein		
			Whole Muscle	Nucleus	ARE-Binding
Li et al., [41]	AE	—	—	↑	↑
Crilly et al., [17]	AE TR	— —	— ↓	↑ —	↑ —
Merry et al., [36]	AE TR	↑ ↑	— —	— —	— —
Wang et al., [40] *	AE	↑	↑	—	—
Mei et al., [45]	TR	↑	↔	—	—
Yamada et al., [42]	AE TR	— —	— ↑	↑ —	↑ —

↑: Increased, ↓: Decreased, ↔: Unchanged vs. age-matched wild-type mice, —not reported. *: examined using ICR/CD-1 mice, while C57/BL6 mice were used in all of the unmarked references. Abbreviations: ARE, antioxidant response element; AE, acute exercise; TR, training.

**Table 4:** Effects of Nrf2 deficiency on skeletal muscle function in C57/BL6 mice.

Characteristics	Animal Age	
	2–8 Months	20–24 Months
Muscle mass	↔ [17,20]	↓ [22,23]
Cross sectional area	↔ [34,53,54]	↓ [18,34]
Grip strength	↔ [34,42]	↓ [22,34]
Running capacity Voluntary wheel running Incremental treadmill running	↔ [17,42] ↓ [35,53], ↔ [17,34,42]	— ↓ [34]
In situ/in vitro contractility	↓ [17,35]	↓ [22]

↑: Increased, ↓: Decreased, ↔: Unchanged vs. age-matched wild-type mice, —not reported. The numbers in brackets are reference numbers.
